# Complement inhibition can decrease the haemostatic response in a microvascular bleeding model at multiple levels

**DOI:** 10.3389/fimmu.2023.1226832

**Published:** 2023-09-13

**Authors:** Murielle Golomingi, Jessie Kohler, Christina Lamers, Richard B. Pouw, Daniel Ricklin, József Dobó, Péter Gál, Gábor Pál, Bence Kiss, Arthur Dopler, Christoph Q. Schmidt, Elaissa Trybus Hardy, Wilbur Lam, Verena Schroeder

**Affiliations:** ^1^ Experimental Haemostasis Group, Department for BioMedical Research (DBMR), University of Bern, Bern, Switzerland; ^2^ Molecular Pharmacy Group, Department of Pharmaceutical Sciences, University of Basel, Basel, Switzerland; ^3^ Institute of Enzymology, Research Centre for Natural Sciences, Budapest, Hungary; ^4^ Department of Biochemistry, Eötvös Loránd University, Budapest, Hungary; ^5^ Institute of Experimental and Clinical Pharmacology, Toxicology and Pharmacology of Natural Products, University of Ulm Medical Center, Ulm, Germany; ^6^ Wallace H. Coulter Department of Biomedical Engineering, Georgia Institute of Technology, Atlanta, GA, United States; ^7^ Aflac Cancer and Blood Disorders Center of Children’s Healthcare of Atlanta, Atlanta, GA, United States; ^8^ Department of Pediatrics, Emory University, Atlanta, GA, United States

**Keywords:** haemostasis, complement system, MBL-associated serine protease-2 (MASP-2), complement C1s, complement factor D (FD), complement C3, complement C5, microfluidics

## Abstract

**Background:**

Haemostasis is a crucial process by which the body stops bleeding. It is achieved by the formation of a platelet plug, which is strengthened by formation of a fibrin mesh mediated by the coagulation cascade. In proinflammatory and prothrombotic conditions, multiple interactions of the complement system and the coagulation cascade are known to aggravate thromboinflammatory processes and increase the risk of arterial and venous thrombosis. Whether those interactions also play a relevant role during the physiological process of haemostasis is not yet completely understood. The aim of this study was to investigate the potential role of complement components and activation during the haemostatic response to mechanical vessel injury.

**Methods:**

We used a microvascular bleeding model that simulates a blood vessel, featuring human endothelial cells, perfusion with fresh human whole blood, and an inducible mechanical injury to the vessel. We studied the effects of complement inhibitors against components of the lectin (MASP-1, MASP-2), classical (C1s), alternative (FD) and common pathways (C3, C5), as well as a novel triple fusion inhibitor of all three complement pathways (TriFu). Effects on clot formation were analysed by recording of fibrin deposition and the platelet activation marker CD62P at the injury site in real time using a confocal microscope.

**Results:**

With the inhibitors targeting MASP-2 or C1s, no significant reduction of fibrin formation was observed, while platelet activation was significantly reduced in the presence of the FD inhibitor. Both common pathway inhibitors targeting C3 or C5, respectively, were associated with a substantial reduction of fibrin formation, and platelet activation was also reduced in the presence of the C3 inhibitor. Triple inhibition of all three activation pathways at the C3-convertase level by TriFu reduced both fibrin formation and platelet activation. When several complement inhibitors were directly compared in two individual donors, TriFu and the inhibitors of MASP-1 and C3 had the strongest effects on clot formation.

**Conclusion:**

The observed impact of complement inhibition on reducing fibrin clot formation and platelet activation suggests a role of the complement system in haemostasis, with modulators of complement initiation, amplification or effector functions showing distinct profiles. While the interactions between complement and coagulation might have evolved to support haemostasis and protect against bleeding in case of vessel injury, they can turn harmful in pathological conditions when aggravating thromboinflammation and promoting thrombosis.

## 1 Introduction

The complement system is part of the innate immune system. While mostly known as the process which leads to the formation of the membrane attack complex (MAC) that disrupts the cell membrane of target cells to trigger cell lysis and death, the complement system exerts additional effector functions, such as opsonisation of targeted cells and promotion of inflammation (1, 2). Haemostasis on the other hand is the process that leads to termination of bleeding from an injured blood vessel. It is initiated in three major steps: vasoconstriction, platelet plug formation and fibrin clot formation mediated by the coagulation cascade (3). Both the complement system and coagulation cascade rely on the sequential activation of serine proteases and require the presence of foreign or altered surfaces to be activated and provide an innate defence against external threats.

As summarised in numerous reviews (4–6), there is extensive crosstalk between the complement and coagulation system, which is not surprising since they share a common evolutionary origin (7). For example, complement components such as C3, C4, C5a and factor B (FB) are found in thrombi (8) and we previously showed that mannose-binding lectin (MBL) of the lectin pathway (LP) of complement activation co-localises with activated platelets and von Willebrand factor (vWF) in a microvascular bleeding model (9). MBL-associated serine proteases 1 and 2 (MASP-1, MASP-2) of the lectin pathway have been shown to bind to activated platelets (10) and C3 to bind vWF (11). Moreover, the alternative complement pathway (AP) has been shown to assemble and activate on ultra-large vWF multimeric strings anchored on endothelial cells (12). We previously showed that MASP-1 can activate prothrombin (13) and that inhibition of MBL and MASP-1 reduces fibrin formation and/or platelet activation at the injury site in a microvascular bleeding model (9).

Activation of the complement and coagulation cascades also leads to the activation of blood cells and endothelial cells and results in a proinflammatory and prothrombotic condition termed thromboinflammation (14). Thromboinflammation is a feature of many diseases, including sepsis, diabetes, autoimmune and cardiovascular diseases.

Furthermore, in complement-mediated diseases, such as paroxysmal nocturnal haemoglobinuria (PNH) or atypical haemolytic uremic syndrome (aHUS), thrombosis is a common complication (14, 15). Studies have suggested that the disruption of platelets by the MAC is an important contributor in the development of thrombosis by inducing the formation of phosphatidylserine-exposing micro-vesicles, which then trigger the formation of the prothrombinase complex (16, 17). Another pathology that involves complement activation and is associated with an increased thrombotic risk is cold agglutinin disease (CAD) (18). In CAD, autoantibodies bind to erythrocytes which leads to classical pathway (CP) activation (19). During the past two decades, several complement-targeted therapeutics have been approved or reached late-stage clinical development, with other candidates listed in the pipelines of pharmaceutical companies (20). Eculizumab (21), an anti-C5 antibody that blocks the formation of C5a and MAC, was the first complement-specific drug approved for the treatment of PNH and aHUS, with later expansion to other rare indications. Pegcetacoplan, a C3 inhibitor of the compstatin family (22), marked the second inhibitor class for PNH therapy, whereas the anti-C1s antibody sutimlimab (23) is used for treatment of CAD.

When investigating crosstalk between complement and coagulation, many studies were performed using blood plasma/serum or purified proteins. While easy to perform and suitable for specific mechanistical aspects, critical factors that influence complement and coagulation are missing in such settings; this includes blood flow, other blood cells and components and the presence of the endothelium. Animal studies can be used to perform experiments under physiological conditions, but they do not always reflect human physiology, are ethically questionable and often difficult to perform. To obtain results that are closer and more relevant to human physiology, we employ a microvascular bleeding model. This model consists of an artificial blood vessel made of human endothelial cells that are embedded in an organosilicon chip and perfused with human whole blood. The term “microvascular” refers to the diameter of the artificial vessels (50 µm) that corresponds to human venoles, even though human umbilical vein endothelial cells (HUVECs) of macrovascular origin were used. The blood vessel can be mechanically injured, simulating a small vessel injury and leading to the formation of a blood clot, which can be monitored in real time with a confocal microscope (24).

Even though the last few decades of research provided clear evidence for close interactions between complement and coagulation, a better understanding of the relevance and (patho-) physiological consequences of the crosstalk between the two systems is required. In particular, while such interactions have been mainly studied in the context of thromboinflammation, it is not known if and how the two systems interact in physiological situations without underlying proinflammatory and prothrombotic conditions. Therefore, the aim of this study was to investigate effects of the complement system on the normal physiological haemostatic response upon vascular injury using a microvascular bleeding model. We studied the effects of specific inhibitors targeting individual or multiple pathways of the complement system on fibrin formation and platelet activation at the injury site to determine if, – and which parts of –, complement activation may play a role in haemostasis.

## 2 Materials and methods

### 2.1 Preparation of the microfluidic bleeding model device

The bleeding model devices were prepared from polydimethylsiloxane (PDMS) as described before (25). The seeding of endothelial cells into the microvascular bleeding model was performed as described before (9). Briefly: The devices were first coated with a collagen solution (rat tail collagen I diluted in ddH_2_O; Thermofisher, Switzerland), followed by a fibronectin solution (0.005%; Merck). Confluent P5 HUVEC (Lonza, Switzerland) cells were trypsinised and spun down. The cell pellet was resuspended in a dextran solution (80 µg/ml diluted in cell culture medium) and filtered through a cell strainer (35 µM; Corning, USA). Approx. 4x10^5^ cells in a dextran suspension were injected into the main channel of each device. The seeded devices were incubated for an hour before being connected to a syringe containing cell culture medium and perfused for 48 h at an initial flow rate of 1 µl/min for 12 h and 2 µl/min for the remaining 36 h to grow to a confluent monolayer of endothelial cells.

### 2.2 Bleeding experiments

The bleeding experiments were performed as described before (9). Briefly: On the day of the experiment, the cells grown in the device were stained with Cellmask™ Orange plasma membrane stain (1 µl/ml; Thermofisher). The device was then installed on the confocal microscope stage (Zeiss LSM 710 with Airyscan). A 2 ml vacuum was created on the valve channel. Freshly drawn citrated whole blood from anonymous healthy blood donors (purchased from the blood donation centre Bern, Switzerland) was supplemented with 40 µg/ml corn trypsin inhibitor (CTI; Loxo GmbH, Germany) to prevent technical interferences by clotting via the contact pathway of coagulation; the extrinsic pathway is not affected by this procedure. Fluorescently labelled platelet antibody and fibrinogen, as well as complement inhibitors as detailed below. The time between addition of the complement inhibitors and start of the experiment was around 30 min.The blood sample was recalcified (final concentration 12.5 mM) and used to perfuse the main channel at a flow rate of 2 µl/min. Pressure was exerted via the side channel to create an injury to the main channel and the recording of images of the injury site (1 image per min for 40 min) was started.

### 2.3 Fluorescence intensity measurements to quantify clot formation

**Figure 1:**
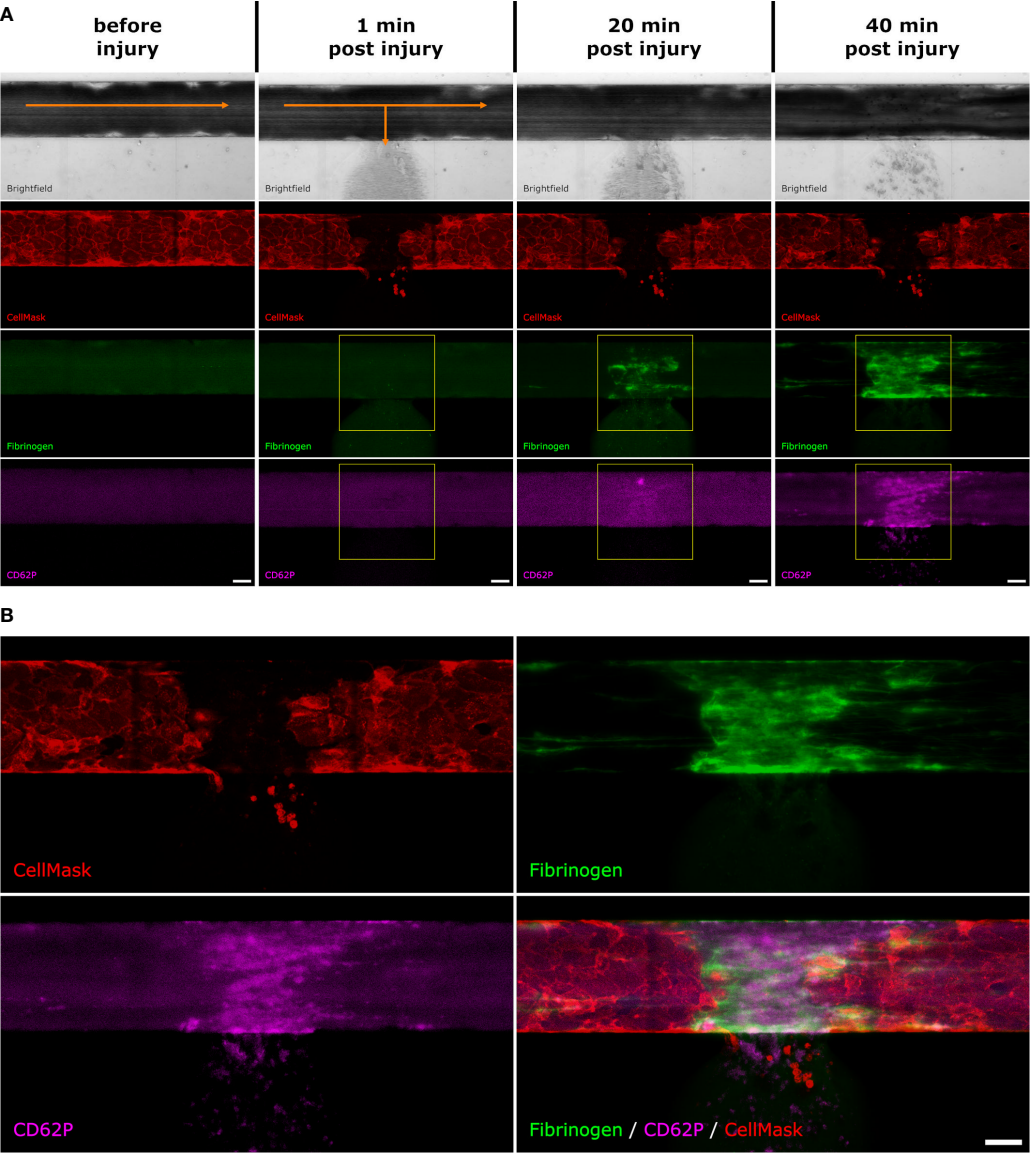
Fluorescence intensity measurements. After the artificial vessel was stained with CellMask and the whole blood was supplemented with fluorescently labelled fibrinogen as well as anti-CD62P antibodies, the microvascular model was placed under the microscope. **(A)** Before injury: In the brightfield channel, the blood is flowing from left to right (indicated with the orange arrow). The endothelial cell membranes are stained with CellMask (red). There is a slight background signal of the labelled fibrinogen (green) and CD62P (pink). 1 min post injury: directly after injury, the blood starts to flow out towards the side channel (indicated with the orange arrows). The fibrin (green) and CD62P (pink) signals start to appear. The yellow square shows the ROI which was selected to measure the fluorescence intensity of the fibrin and CD62P signal. 20 min post injury: After 20 min, the fibrin (green) and CD62P (pink) signals become stronger. The yellow square shows the ROI which was selected to measure the fluorescence intensity. 40 min post injury: After 40 min the experiment is stopped. **(B)** After 40 min, fibrin (green) and CD62P (pink) are both present where the endothelial layer (red) was injured. Scale bar: 50 µm.

Clot formation was detected and quantified by measuring fibrin formation and platelet activation. To detect fibrin formation, the blood samples were supplemented with a fibrinogen-AlexaFluor™647-conjugate (10µl/600µl of blood, Invitrogen F35200; Thermofisher). To detect platelet activation, we used a polyclonal sheep anti-CD62P (P-selectin) primary antibody, (10µl/600µl of blood; R&D Systems, Inc. AF137, Bio-Techne) and an AlexaFluor™647-labelled secondary donkey anti-sheep antibody (0.75µl/600µl of blood; ab150179, Abcam, UK). The relative fluorescence signal intensities were measured over time in a region of interest (ROI) of 300x300 µm^2^ around the injury site as shown in **Figure 1**. The area under the curve (AuC) was calculated with the software Prism with the baseline set to 0 in these calculations.

### 2.4 Complement inhibitors

Freshly drawn whole blood samples from healthy donors were supplemented with either one of the following complement inhibitors or with PBS as control. Surrogates of the clinical antibodies sutimlimab (anti-C1s; AH803), lampalizumab (anti-factor D (FD); AA662) and eculizumab (anti-C5; AA552) with specific activities for the CP, AP, and terminal complement pathway, respectively, were expressed as scFv_2_-Fc (IgG1) ‘minibody’ format by the Geneva Antibody Facility (https://www.unige.ch/medecine/antibodies/). A LP inhibitor that blocks MASP-2 (SGMI-2) was recombinantly expressed and the C3 inhibitor compstatin Cp20 was produced by peptide synthesis as described before (26, 27). To achieve a strong inhibition of each target, we used inhibitor concentrations that were at least 2.5 times higher than the normal plasma concentration of the corresponding complement components reported in literature (where applicable) (**Table 1**). In addition, we used the triple inhibitor TriFu (triple-fusion-inhibitor), which is based on the three different regulatory proteins DAF (1–4)-FH (19, 20)-CR1 (15–17) and inhibits C3b generation through all three complement pathways (35). TriFu was used at a final concentration of 2 µM. For a comparative experiment, we included the MASP-1-specific inhibitor SGMI-1 which significantly reduced fibrin formation and platelet activation in our recent study (9); SGMI-1 was produced as described before (27).

**Table 1:** Complement components that were inhibited in this study and their molecular weight (MW), normal mass and molar concentration in blood, their corresponding inhibitors and final inhibitor concentration used in the experiments.

Complement component	Reported MW	Reported mass concentration in serum or plasma	Calculated molar concentration	Inhibitor used	Final concentration of inhibitor in blood sample
C1s	75 kDa (28)	34 µg/ml (29)	453 nM	Anti-C1s inhibitory antibody	1135 nM
MASP-2	76 kDa (30)	0.4 μg/ml (31)	5.3 nM	SGMI-2	164.5 nM
Factor D	24 kDa (32)	3 µg/ml (29)	125 nM	Anti-FD inhibitory antibody	307 nM
C3	195 kDa (33)	1200 µg/ml (29)	6.2 µM	Cp20	16.3 µM
C5	188 kDa (34)	50 µg/ml (29)	266 nM	Anti-C5 inhibitory antibody	925 nM

## 3 Results

### 3.1 Inhibition of individual complement activation pathways

The initiation of the complement system on injured host cells can be mediated via three distinct pathways that recognize damage-associated molecular patterns. Whereas the CP and LP are triggered by antibody clusters and carbohydrate signatures on cell surfaces, respectively, the AP is constitutively activated at low level, can be induced by various events and drives the amplification of the complement response. The use of pathway-specific inhibitors in our microvascular bleeding model may therefore provide insight into the targets and processes involved in physiological complement-coagulation crosstalk.

**Figure 2:**
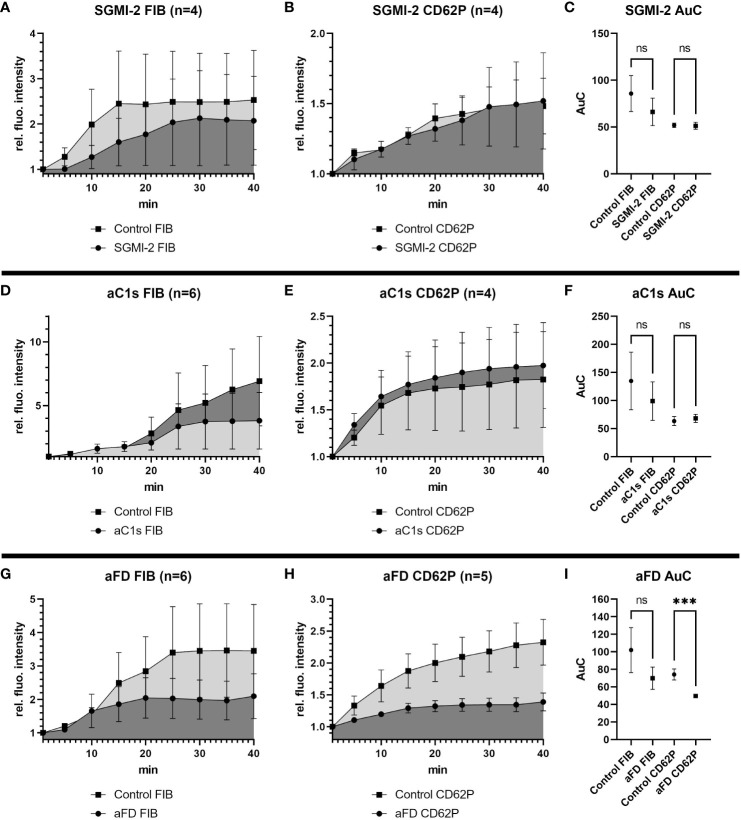
Effects of inhibition of MASP-2, C1s or Factor D in the bleeding model. To investigate the effects of the different complement inhibitors and inhibitory antibodies, the signal intensity relative to the first time point was monitored for activated platelets (CD62P) and fibrinogen/fibrin. To compare the curves, the Area under the Curve (AuC) was calculated. **(A–C)** Inhibition of MASP-2 with SGMI-2 (164 nM) resulted in no significant difference in fluorescent intensity. **(D–F)** The use of inhibitory antibody against C1s (1135 nM) resulted in no significant difference in fluorescent intensity. **(G–I)** The use of inhibitory antibodies against Factor D (307 nM) resulted in a significant reduction of CD62P signal and statistically non-significant reduction of fibrinogen/fibrin signal. Data are shown as mean with SEM, and p-values for differences between control and inhibitor groups were determined with paired t-test (*p ≤ 0.05, **p ≤ 0.01, ***p ≤ 0.001). ns, not significant.

Targeting the LP with the MASP-2 inhibitor SGMI-2 had no significant effect on fibrin formation or platelet activation, as measured by monitoring relative fluorescence intensity over time of the fibrin and CD62P signal, respectively (**Figures 2A, B**). Even though fibrin formation seemed slightly reduced in the presence of SGMI-2, it was not statistically significant, as shown by comparison of the area under the curve (AuC) of the control experiments without inhibitor (**Figure 2C**).

Similarly, inhibition of the CP using an anti-C1s minibody surrogate of sutimlimab did not lead to statistically significant effects, even if a slightly reduced fibrin and slightly increased CD62P signal was observed (**Figures 2D–F**).

In contrast, when we impaired the AP with an anti-FD minibody (lampalizumab surrogate), a reduction of fluorescence intensity was detected for both fibrin and CD62P; however, due to a high variability in the fibrin signal, only the reduction of the CD62P signal was statistically significant (**Figures 2G–I**). Since inhibition of FD reduced platelet activation upon vascular injury, the AP may support platelet activation during haemostasis.

### 3.2 Broader inhibition of the complement activation and effector generation reduces fibrin formation and platelet activation upon vessel injury

**Figure 3:**
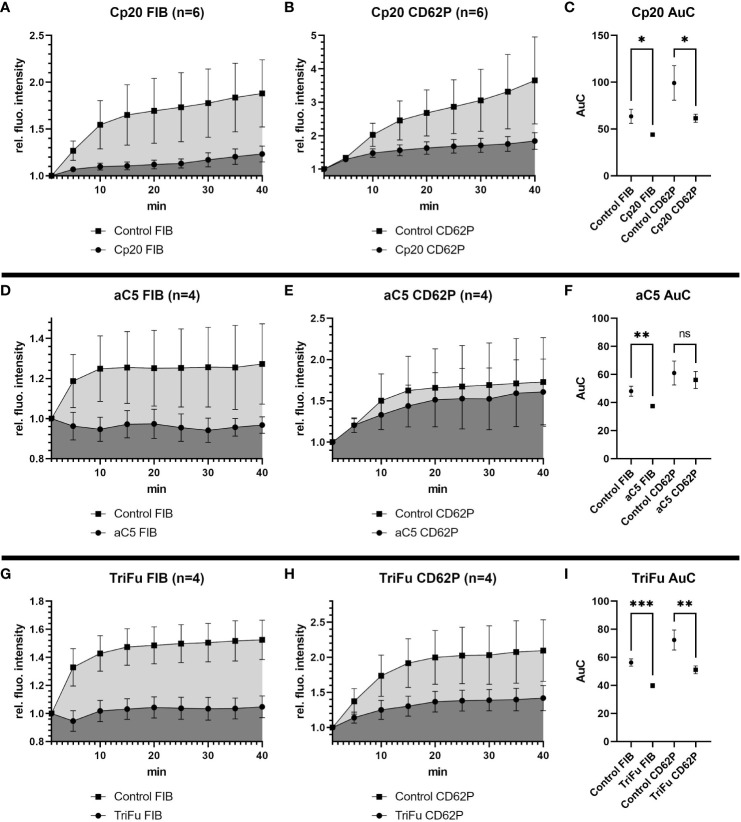
Effects of inhibiting C3, C5 or all complement convertases in the bleeding model. To investigate the effects of the different complement inhibitors and inhibitory antibodies, the signal intensity relative to the first time point was monitored for activated platelets (CD62P) and fibrinogen/fibrin. To compare the curves, the Area under the Curve (AuC) was calculated. **(A–C)** Inhibition of C3 with Cp20 (16.3 µM) resulted in significant reduction of fibrinogen/fibrin and CD62P signal. **(D–F)** The use of inhibitory antibodies against C5 resulted in no significant difference in CD62P signal and significant decrease in fibrinogen/fibrin signal. **(G–I)** Downregulating all complement convertases with the fusion protein TriFu (2 µM) resulted in a significant decrease of fibrinogen/fibrin and CD62P signal. Data are shown as mean with SEM, and p-values for differences between control and inhibitor groups were determined with paired t-test (*p ≤ 0.05, **p ≤ 0.01, ***p ≤ 0.001). ns, not significant.

Under most physiological conditions, complement initiation via either of the three pathways leads to the formation of convertase complexes that activate the plasma proteins C3 and C5 to generate various effector molecules. We therefore targeted these common complement pathways at the level of C3 and C5. Addition of Cp20, a peptidic C3 inhibitor of the compstatin family, resulted in a statistically significant reduction of both the fibrin and CD62P signals (**Figures 3A–C**). Similarly, C5 inhibition using a minibody surrogate of eculizumab resulted in a reduced intensity for both the fibrin and CD62P signals (**Figures 3D–F**), yet only the difference in fibrin was statistically significant. These results indicate that the common pathway of complement might play a role during haemostasis by supporting fibrin formation and platelet activation.

The stronger impact of impairing complement activation more broadly rather than pathway-specifically was also confirmed by the inhibitor TriFu, a cell-targeted complement regulator construct that acts on convertase complexes and opsonins generated via all initiation pathways. Similar to C3 inhibition, the fibrin and CD62P signals were both strongly reduced in presence of TriFu and the difference to the control experiments were statistically significant (**Figures 3G–I**). Together, these results suggest that global complement inhibition at the level of C3/C5 activation may have relevant anticoagulant effects.

### 3.3 Donor-specific comparison of complement inhibition strategies

**Figure 4:**
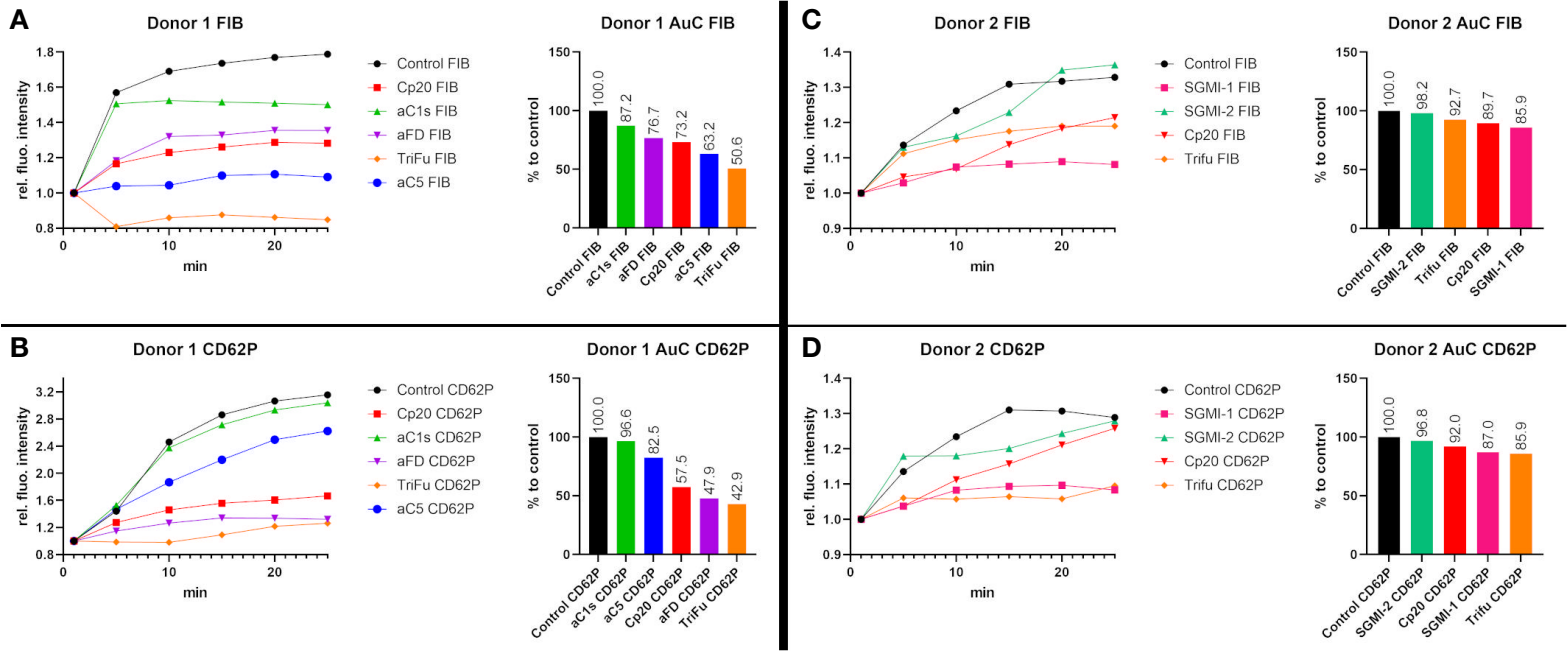
Direct comparison of several complement inhibitors in two individual blood donors. To investigate the effects of the different complement inhibitors and inhibitory antibodies, two different series of experiments were performed using the same blood samples. **(A, B)** The blood sample of donor 1 was used to compare the effect of Cp20 (16.3 µM), the inhibitory antibody against C1s (1135 nM), the inhibitory antibody against FD (307 nM), TriFu (2 µM) and the inhibitory antibody against C5 (925 nM). The signal intensity relative to the first time point was monitored for activated platelets (CD62P) and fibrinogen/fibrin (FIB). To compare the curves, the Area under the Curve (AuC) was measured and the AuC in comparison to the control was calculated. **(C, D)** The blood sample of donor 2 was used to compare the effect of Cp20 (16.25 µM), TriFu (2 µM), SGMI-1 (5 μM) and SGMI-2 (164.5 nM). The signal intensity relative to the first time point was monitored for activated platelets (CD62P) and fibrinogen/fibrin (FIB). To compare the curves, the Area under the Curve (AuC) was measured and the AuC in comparison to the control was calculated.

Our results led to the question, which complement inhibitors may exert the biggest effects on fibrin formation and platelet activation. We therefore performed two exploratory experiments where we tested and directly compared several inhibitors in the blood from the same individual donors. For technical reasons, we were not able to compare all inhibitors in the same experiment; we therefore included TriFu and inhibitors of C1s, FD, C3, and C5 in the sample from donor 1 (**Figures 4A, B**) and TriFu and inhibitors of MASP-1, MASP-2, and C3 in the sample from donor 2 (**Figures 4C, D**).

In donor 1, Cp20, anti-C5 and TriFu showed the largest reduction in fibrin formation, while platelet activation was strongly reduced by anti-FD, Cp20 and TriFu. In donor 2, inhibition of MASP-1 and C3, as well as TriFu showed the largest effects, even though to a lesser extent than in donor 1. Overall, these results of direct comparison are consistent with the results described above. They indicate that inhibition at the level of C3, either by acting on the C3 substrate (via Cp20) or convertases (via TriFu), has a profound effect on clot formation. Despite both acting on the LP, inhibition of MASP-1 had a much more pronounced impact when compared to MASP-2 blockage, which may be explained with the broad crosstalk activities reported for MASP-1.

While informative and in agreement with the inhibitor-specific experiments, it has to be noted that interindividual differences and technical variability must be taken into account for the comparative study and additional studies will be needed to corroborate and extend those findings.

## 4 Discussion

The crosstalk between the coagulation cascade and the complement system has raised increasing interest in recent years, in particular in the context of thromboinflammation. The involvement of complement in cardiovascular diseases and their thrombotic manifestations has therefore been the focus of several studies (36). The similarities of complement and coagulation include the fact that both systems rely on a cascade of serine proteases (37) and can be activated by stimuli of altered-self or foreign surfaces.

However, the relevance of the interactions between complement and coagulation under non-pathological conditions is less well understood. Studies on the interaction of different components have often been performed in purified systems, which do not reflect the physiological conditions of haemostasis. The aim of this study was to understand the potential role of complement in the haemostatic response under more physiological conditions. The use of the bleeding model allowed us to observe clot formation in real-time in the context of an injured endothelialised artificial vessel and whole blood under flow conditions. While state of the art, the current bleeding model that uses freshly drawn whole blood also imposes limitations regarding the number of possible experiments. We therefore carefully selected the panel of tested inhibitors to include molecules with well-described activity and selectivity profiles and high clinical significance. In this context, it must be noted that while the anti-C1s, -FD, and -C5 minibodies used in this study share the antigen-binding sequence with sutimlimab, lampalizumab and eculizumab, respectively, they do otherwise not correspond to the clinical antibody products. In future experiments, it would be interesting to include inhibitors against other complement targets or complement regulators such as factor H (FH) to investigate and compare their effects on the haemostatic response. Moreover, it would also be interesting to apply mono-specific inhibitors in combinations to see if their effects are additive or synergistic.

### 4.1 Modulation at the level of complement initiation

The initiation of complement activation via pattern recognition complexes of the CP and LP upon sensing of pathogen- and damage-associated molecular patterns is a hallmark of complement-mediated host defense and homeostasis. The CP is activated when the C1 complex, consisting of the pattern recognition protein C1q associated with zymogen forms of the serine proteases C1r and C1s, binds to the Fc region of antibody clusters on cell surfaces (38) but also to bacterial and viral surface proteins, apoptotic cells, and acute phase proteins (39). The binding leads to a sequential activation of C1r and C1s, latter of which cleaves complement component C2 and C4 to form C3 convertases. There are studies connecting the CP with coagulation; for example, platelets have receptors for C1q that were reported to induce the expression of integrins and CD62P, which may amount to a procoagulant effect (40). Clinical conditions involving CP activation, such as cold agglutinin disease (CAD), can also lead to an increased risk for thrombotic events (18). However, there is no indication that C1s can activate prothrombin in the same manner as MASP-1 of the lectin pathway (41).

So far, the only approved therapeutic specific for the CP is sutimlimab for treatment of CAD. Sutimlimab is a humanised monoclonal antibody which inhibits activation of C1s of the CP without blocking the opsonic function of C1q (42). Treatment of CAD patients with sutimlimab showed decreased levels of D-dimer and thrombin–antithrombin complex during treatment (23, 43), indicating an effect of C1s inhibition on coagulation. When we used a minibody with the same paratope as sutimlimab in the microfluidic bleeding model, we observed a notable yet not significant reduction of the relative fluorescence signal of fibrinogen at the injury site, but no difference in the CD62P signal. This apparent discrepancy may indicate that activation of the CP may have a relevant procoagulant effect under pathophysiological conditions (e.g., in CAD) but is of less significance during non-pathological haemostasis, possibly because there are no or not sufficient stimuli to activate the CP.

Similar to the CP, LP activation involves complexes of pattern recognition proteins, including MBL, ficolins and collectins, with associated serine proteases (i.e. MASP-1 and MASP-2). Binding of the complex to carbohydrate patterns on the surface of microorganisms or injured host cells (44, 45) leads to the sequential activation of MASP-1 and MASP-2 to initiate the formation of C3 convertases. There is a plethora of studies about the interactions of the LP with coagulation. MBL, MASP-1 and MASP-2 were shown to bind to fibrin (46), and MASP-1 and -2 were shown to bind to activated platelets (10). In a previous study, we showed that MBL and MASP-1 are present at the injury site in the microvascular bleeding model (9). While there was no clear co-localisation with fibrinogen, MBL co-localised with vWF and activated platelets. Studies also showed that MASP-1 and -2 can cleave prothrombin and fibrinogen and can activate Factor XIII of the coagulation cascade (13, 41, 47–49). We also demonstrated that MASP-1 contributes to platelet activation via thrombin generation (9). The thrombin-like activity of MBL-MASP complexes is not detectable in MASP-1 KO serum but is still present in MASP-2 deficient serum (50). In agreement, MBL and MASP-1/3 KO mice show a prolonged bleeding time, in comparison with WT and MASP-2 KO mice (48). Blocking of MBL with the monoclonal antibody 3F8 in a mouse model decreased infarct size and fibrin deposition after myocardial ischemia/reperfusion and prevented ferric chloride–induced occlusive arterial thrombogenesis (51). In our recent study, we used the same inhibitory antibody against MBL and a selective MASP-1 inhibitor in the microvascular bleeding model and showed that blocking of MBL leads to a decrease of platelet activation at the injury site and reduced MBL and MASP-1 deposition. Inhibition of MASP-1 leads to a decrease in platelet activation and fibrin formation (9). All these studies point towards procoagulant effects of the lectin pathway.

In this study, we used the *Schistocerca gregaria* protease inhibitor (SGPI)-based MASP-2 inhibitor SGMI-2 (27) in the microvascular bleeding model and measured fibrin formation and platelet activation at the injury site. While we saw a decrease in both fibrin formation and platelet activation, the difference to the control was not statistically significant. In a previous study using a rotation-thromboelastometry system (ROTEM), we showed that inhibition of MASP-2 did not lead to any changes in clotting time or clot formation time (13). Our results confirm the procoagulant effect of MASP-1, both in pathological settings and during physiological haemostasis. As blockage of MASP-2 did not result in a notable effect on clot formation or platelet activation, it remains to be determined whether the impact of MASP-1 is linked to LP activation or achieved independently. The LP might function as a redundant system to support haemostasis by recognising vascular injury with MBL and then amplifying platelet activation and fibrin formation with MASP-1 in a thrombin-dependent manner.

### 4.2 Modulation at the level of C3 and C5 activation

Although the AP can be triggered independently of the CP and LP, via low level tick-over or surface interactions, it also provides the main mechanism of complement amplification and effector generation. Deposition of the C3b opsonin by any pathway leads to the formation of AP C3 convertase complexes when FB binds to C3b and is activated by FD. As the resulting C3 convertases generate and deposit more C3b on the surface, this leads to continuous opsonization and amplification of the complement response. While C3b and its degradation products act as effector molecules themselves by interacting with complement receptors on immune cells, increasing C3b densities also lead to the formation of convertases that preferentially activate C5. The cleavage of C5 generates the potent anaphylatoxin C5a and induces MAC formation that can damage or lyse cells. Due to the central role of the AP and convertase-mediated C3 and C5 activation, these processes are tightly controlled by physiological regulators such as FH and a main target for complement therapeutics.

There are links between AP activation and coagulation. For example, in diseases involving excessive AP activation such as PNH, the risk of thrombosis is increased (15). PNH is caused by an absence of the AP regulators CD55 and CD59 on the surface of erythrocytes and platelets, which leads to poorly controlled complement activation with lysis of cell membranes. While the mechanism of thrombosis in PNH is not fully elucidated, therapeutic complement inhibition results in a decreased thrombotic risk for PNH patients (52). In a mouse model of aHUS, based on a FH mutation found in humans, the mice suffered from diffuse microvascular and macrovascular thrombosis, while the lack of FD in those mice prevented thrombosis (53, 54). Studies also showed that vWF secreted from endothelial cells serves as a nucleus for the assembly of AP components (12), and that vWF can directly interact with FH (12, 55). Another study showed that the inhibition of FD in heparinised human whole blood inhibited platelet activation in a simulated cardiopulmonary bypass circuits (56). All these studies presented evidence that the AP plays a role in thrombosis.

The critical role of FD in the formation of the AP C3 convertase, but not other C3 convertases, and its rather low plasma concentration render FD an interesting target for AP-specific inhibitors. In this study, we used a minibody surrogate of lampalizumab, and antibody that was developed to treat patient with geographic atrophy, an advanced form of age-related macular degeneration, but was discontinued after phase 3 trials (57). We found that inhibition of FD decreased both fibrin formation and platelet activation at the injury site. However, only the difference in platelet activation was statistically significant. Our results indicate that the AP, or FD, may play a role in haemostasis, mainly by increasing platelet activation. The observed reduction in the CD62P signal in our bleeding model upon FD inhibition may be explained by the finding that AP components are assembled and activated on EC-secreted/anchored ULVWF multimeric strings (12). Without FD, the AP C3 convertase cannot form and there is no release of C3a, which was shown to activate platelets (58).

The activation of C3 by any of the C3 convertases marks a central and converging step in the complement response, which largely defines the generation of complement effectors and mediators. C3 is among the most highly concentrated proteins in circulation but remains functionally inert before it binds to C3 convertases. Once convertases are formed, C3 is cleaved into the opsonin C3b, which enables signaling via complement receptors and fuels the formation of additional C3 and C5 convertases. Cleavage of C5, on the other hand, generates C5b as initiator of MAC formation. Both activation events liberate anaphylatoxins (C3a, C5a), which exert potent chemoattractive, inflammatory and immune-modulatory functions.

The common pathway, defined by convertase-mediated C3 and C5 activation, has been shown to influence coagulation. The anaphylatoxins C3a and C5a induce upregulation of leukocyte adhesion molecules required for coagulation such as P-selectin (CD62P), vWF and TF on endothelial cells (59). Moreover, C5a and C3a have been reported to activate platelets and trigger their aggregation (58, 60), although recent studies suggest that C3-dependent platelet aggregation is induced by C3b rather than C3a (61). In line with this another recent study demonstrated that anaphylatoxins C3a and C5a do not directly act on platelets but rather influence platelet activation indirectly via the activation of leukocytes (62). C3 is present in thrombi, affects clot structure and increases resistance to fibrinolysis (8). Mice lacking the C3 gene have delayed thrombotic response and their thrombi are less stable. Interestingly, these mice have longer bleeding time which was attributed to subnormal platelet function (63). C3 was also shown to bind to vWF (11), one of the major components of haemostasis.

In this study, we used several inhibitors that interfere in the C3-C5 axis, yet via distinct mechanisms. The C3 inhibitor Cp20 is a member of the compstatin family (26), which also includes the clinical therapeutic pegcetacoplan that is meanwhile approved for the treatment of PNH and geographic atrophy and the candidate drug AMY-101 (Cp40) (20, 22). While Cp20 mainly acts by preventing the binding of C3 to the convertases, the regulator-based TriFu interferes with convertase formation and opsonization. It combines inhibitory domains of the complement regulators CD35 (CR1) and CD55 (DAF) with the C-terminal domains of FH, latter of which enables a preferred targeting for host cells (64, 65). Although distinct in their mode of action, both Cp20 and TriFu result in a broad control of complement activation. Indeed, both inhibitors demonstrated a similar profile with potent effects on both fibrin deposition and platelet activation, thereby supporting the hypothesis that C3 promotes clot formation and thereby plays a role in haemostasis.

As C5 activation is located downstream of C3 activation, it is not clear whether the effects observed with Cp20 and TriFu are primarily induced by preventing the formation of C3- or C5-derived effectors. In patients with PNH, the disruption of endothelial cells and platelets by the MAC results in production of microvesicles that expose phosphatidylserine, which allows the formation of the prothrombinase complex (16, 17). The anti-C5 antibody eculizumab was the first complement-specific therapeutic (21); while initially approved for the treatment of PNH, its indications meanwhile include aHUS, myasthenia gravis and neuromyelitis optica-related disorders. Eculizumab treatment leads to reduced coagulation activation markers, which indicates a downregulation of the coagulation cascade (21). Using a minibody surrogate of eculizumab in our model, we found that inhibition of C5 reduced both platelet activation and fibrin formation, though only the difference in fibrin formation was significant. Our results indicate that C5 may have a procoagulant effect in haemostasis but it remains to be further investigated how similar or distinct the impact of C3 versus C5 activation is in different haemostatic conditions. Several years of clinical complement inhibition at the levels of C1s, C3 and C5 have not led to any substantial susceptibility to bleeding complications. This indicates that complement may be dispensable for everyday steady state haemostatic responses. However, it would be extremely interesting to see the effect of complement on stopping bleeding in patients who have impaired mechanisms of haemostasis (e.g. due to medication with anticoagulants or loss of coagulation proteins due to consumption after severe trauma or burns etc.).

### 4.3 Overall conclusion

Taken together, our results show that in a human *in vitro* model of bleeding, the complement system as a whole and, in particular, FD of the AP, MASP-1 of the LP, as well as C3 and C5 of the common pathway may support the haemostatic response. Thereby, our findings add to the growing perception that complement, coagulation, platelets and endothelial cells strongly collaborate in host defense and imply that the impact of complement in haemostasis is not restricted to harmful effects through aggravating thromboinflammation but may also act as a protective factor against bleeding in the case of vessel injury. It will be important to further delineate the involvement of complement in haemostasis during physiological and pathophysiological processes in future studies, and to investigate if and how this important crosstalk can be exploited for therapeutic purposes.

## Data availability statement

The original contributions presented in the study are included in the article/**Supplementary Material**. Further inquiries can be directed to the corresponding author.

## Ethics statement

Ethical approval was not required for the studies involving humans because whole blood from anonymous healthy blood donors was purchased from the blood donation centre Bern, Switzerland. The blood donation centre obtained the overall consent of the participants to use some of the blood for research purposes. Therefore, ethics approval and informed consent for our particular study was not required according to Swiss legislation. The studies were conducted in accordance with the local legislation and institutional requirements. The human samples used in this study were acquired from a by- product of routine care or industry. Written informed consent to participate in this study was not required from the participants or the participants’ legal guardians/next of kin in accordance with the national legislation and the institutional requirements.

## Author contributions

MG and JK performed the microfluidic bleeding model experiments. MG performed the statistical analysis. EH and WL designed the microfluidic bleeding model and provided crucial material for this study. JD, PG, GP and BK produced and provided the MASP-1 inhibitor (SGMI-1) and MASP-2 inhibitor (SGMI-2) and contributed to data interpretation. CS and AD produced and provided the fusion-protein TriFu and contributed to data interpretation. CL, RP and DR produced and provided the inhibitory monoclonal antibodies against C1s, FD and C5 as well as the C3 inhibitor Cp20 and contributed to data interpretation. VS designed and supervised the study. MG wrote the first draft of the manuscript. All authors contributed to manuscript revision, read and approved the submitted version. All authors contributed to the article and approved the submitted version.

## References

[1] Ricklin D Hajishengallis G Yang K Lambris JD. Complement: a key system for immune surveillance and homeostasis. Nat Immunol (2010) 11(9):785–97. doi: 10.1038/ni.1923 PMC2924908 20720586

[2] Dunkelberger JR Song W-C. Complement and its role in innate and adaptive immune responses. Cell Res (2010) 20(1):34–50. doi: 10.1038/cr.2009.139 20010915

[3] Gale AJ. Continuing education course #2: current understanding of hemostasis. Toxicol Pathol (2011) 39(1):273–80. doi: 10.1177/0192623310389474 PMC3126677 21119054

[4] Conway EM. Reincarnation of ancient links between coagulation and complement. J Thromb Haemost. (2015) 13(S1):S121–32. doi: 10.1111/jth.12950 26149013

[5] Oikonomopoulou K Ricklin D Ward PA Lambris JD. Interactions between coagulation and complement–their role in inflammation. Semin Immunopathol [Internet]. 2011/08/03. (2012) 34(1):151–65. doi: 10.1007/s00281-011-0280-x PMC3372068 21811895

[6] Keragala CB Draxler DF McQuilten ZK Medcalf RL. Haemostasis and innate immunity – a complementary relationship: A review of the intricate relationship between coagulation and complement pathways. Br J Haematol (2018) 180(6):782–98. doi: 10.1111/bjh.15062 29265338

[7] Dzik S. Complement and coagulation: cross talk through time. Transfus Med Rev [Internet]. (2019) 33(4):199–206. doi: 10.1016/j.tmrv.2019.08.004 31672340

[8] Howes JM Richardson VR Smith KA Schroeder V SOmani R Shore A. Complement C3 is a novel plasma clot component with anti-fibrinolytic properties. Diabetes Vasc Dis Res (2012) 9(3):216–25. doi: 10.1177/1479164111432788 22253322

[9] Golomingi M Kohler J Jenny L Hardy ET Dobó J Gál P. Complement lectin pathway components MBL and MASP-1 promote haemostasis upon vessel injury in a microvascular bleeding model. Front Immunol (2022) 13:948190. doi: 10.3389/fimmu.2022.948190 36032172 PMC9412763

[10] Kozarcanin H Lood C Munthe-Fog L Sandholm K Hamad OA Bengtsson AA. The lectin complement pathway serine proteases (MASPs) represent a possible crossroad between the coagulation and complement systems in thromboinflammation. J Thromb Haemost. (2016) 14(3):531–45. doi: 10.1111/jth.13208 26614707

[11] Nolasco JG Nolasco LH Da Q Cirlos S Ruggeri ZM Moake JL. Complement component C3 binds to the A3 domain of von Willebrand factor. TH Open (2018) 2(3):e338–45. doi: 10.1055/s-0038-1672189 PMC6508891 31080944

[12] Turner NA Moake J. Assembly and activation of alternative complement components on endothelial cell-anchored ultra-large von Willebrand factor links complement and hemostasis-thrombosis. PloS One (2013) 8(3):e59372. doi: 10.1371/journal.pone.0059372 23555663 PMC3612042

[13] Jenny L Dobo J Gal P Schroeder V. MASP-1 of the complement system promotes clotting via prothrombin activation. Mol Immunol (2015) 65(2):398–405. doi: 10.1016/j.molimm.2015.02.014 25745807

[14] Ekdahl KN Teramura Y Hamad OA Asif S Duehrkop C Fromell K. Dangerous liaisons: complement, coagulation, and kallikrein/kinin cross-talk act as a linchpin in the events leading to thromboinflammation. Immunol Rev (2016) 274(1):245–69. doi: 10.1111/imr.12471 27782319

[15] Korkama E-S Armstrong A-E Jarva H Meri S. Spontaneous remission in paroxysmal nocturnal hemoglobinuria-return to health or transition into malignancy? Front Immunol (2018) 9:1749. doi: 10.3389/fimmu.2018.01749 30116241 PMC6082924

[16] Hamilton KK Hattori R Esmon CT Sims PJ. Complement proteins C5b-9 induce vesiculation of the endothelial plasma membrane and expose catalytic surface for assembly of the prothrombinase enzyme complex. J Biol Chem (1990) 265(7):3809–14. doi: 10.1016/S0021-9258(19)39666-8 2105954

[17] Wiedmer T Esmon CT Sims PJ. Complement proteins C5b-9 stimulate procoagulant activity through platelet prothrombinase. Blood (1986) 68(4):875–80. doi: 10.1182/blood.V68.4.875.875 3092889

[18] Broome CM Cunningham JM Mullins M Jiang X Bylsma LC Fryzek JP. Increased risk of thrombotic events in cold agglutinin disease: A 10-year retrospective analysis. Res Pract Thromb Haemost. (2020) 4(4):628–35. doi: 10.1002/rth2.12333 PMC7292660 32548562

[19] Yin W Ghebrehiwet B Peerschke EIB. Expression of complement components and inhibitors on platelet microparticles. Platelets (2008) 19(3):225–33. doi: 10.1080/09537100701777311 PMC2659880 18432523

[20] Lamers C Ricklin D Lambris JD. Complement-targeted therapeutics: An emerging field enabled by academic drug discovery. Am J Hematol (2023) 98 Suppl 4:S82–9. doi: 10.1002/ajh.26875 36755352

[21] Cofiell R Kukreja A Bedard K Yan Y Mickle AP Ogawa M. Eculizumab reduces complement activation, inflammation, endothelial damage, thrombosis, and renal injury markers in aHUS. Blood (2015) 125(21):3253–62. doi: 10.1182/blood-2014-09-600411 PMC4449039 25833956

[22] Hillmen P Szer J Weitz I Röth A Höchsmann B Panse J. Pegcetacoplan versus eculizumab inparoxysmal nocturnal hemoglobinuria. N Engl J Med (2021) 384(11):1028–37. doi: 10.1056/NEJMoa2029073 33730455

[23] Röth A Barcellini W D’Sa S Miyakawa Y Broome CM Michel M. Sutimlimab in cold agglutinin disease. N Engl J Med (2021) 384(14):1323–34. doi: 10.1056/NEJMoa2027760 33826820

[24] Sakurai Y Hardy ET Ahn B Tran R Fay ME Ciciliano JC. A microengineered vascularized bleeding model that integrates the principal components of hemostasis. Nat Commun (2018) 9(1):509. doi: 10.1038/s41467-018-02990-x 29410404 PMC5802762

[25] Hardy ET Sakurai Y Lam WA. Miniaturized vascularized bleeding model of hemostasis. Methods Mol Biol (2022) 2373:159–75. doi: 10.1007/978-1-0716-1693-2_10 34520012

[26] Qu H Ricklin D Bai H Chen H Reis ES Maciejewski M. New analogs of the clinical complement inhibitor compstatin with subnanomolar affinity and enhanced pharmacokinetic properties. Immunobiology (2013) 218(4):496–505. doi: 10.1016/j.imbio.2012.06.003 22795972 PMC3518557

[27] Héja D Harmat V Fodor K Wilmanns M Dobó J Kékesi KA. Monospecific inhibitors show that both mannan-binding lectin-associated serine protease-1 (MASP-1) and -2 Are essential for lectin pathway activation and reveal structural plasticity of MASP-2. J Biol Chem (2012) 287(24):20290–300. doi: 10.1074/jbc.M112.354332 PMC3370211 22511776

[28] UniProt. P09871 · C1S_HUMAN. (2023). Available at: https://www.uniprot.org/uniprotkb/P09871/entry (Accessed 14.04.2023).

[29] Glovsky MM Ward PA Johnson KJ. Complement determinations in human disease. Ann Allergy Asthma Immunol (2004) 93(6):513–23. doi: 10.1016/S1081-1206(10)61257-4 15609759

[30] UniProt. O00187 · MASP2_HUMAN. Available at: https://www.uniprot.org/uniprotkb/O00187/entry (Accessed 14.04.2023).

[31] Thiel S Jensen L Degn SE Nielsen HJ Gál P Dobó J. Mannan-binding lectin (MBL)-associated serine protease-1 (MASP-1), a serine protease associated with humoral pattern-recognition molecules: normal and acute-phase levels in serum and stoichiometry of lectin pathway components. Clin Exp Immunol (2012) 169(1):38–48. doi: 10.1111/j.1365-2249.2012.04584.x 22670777 PMC3390472

[32] UniProt. P00746 · CFAD_HUMAN. (2023). Available at: https://www.uniprot.org/uniprotkb/P00746/entry (Accessed 14.04.2023).

[33] UniProt. P01024 · CO3_HUMAN. (2023). Available at: https://www.uniprot.org/uniprotkb/P01024/entry (Accessed 14.04.2023).

[34] UniProt. P01031 · CO5_HUMAN. (2023). Available at: https://www.uniprot.org/uniprotkb/P01031/entry (Accessed 14.04.2023).

[35] Dopler A Lang S Akilah A Huber-Lang M Schmidt CQ. A potent, targeted, enzyme-like inhibitor of all three complement activation pathways. EMCHD2022. Mol Immunology; (2022) 150, 198.

[36] Markiewski MM Nilsson B Nilsson Ekdahl K Mollnes TE Lambris JD. Complement and coagulation: strangers or partners in crime? Trends Immunol (2007) 28(4):184–92. doi: 10.1016/j.it.2007.02.006 17336159

[37] Krem MM Di Cera E. Evolution of enzyme cascades from embryonic development to blood coagulation. Trends Biochem Sci (2002) 27(2):67–74. doi: 10.1016/S0968-0004(01)02007-2 11852243

[38] Vignesh P Rawat A Sharma M Singh S. Complement in autoimmune diseases. Clin Chim Acta (2017) 465:123–30. doi: 10.1016/j.cca.2016.12.017 28040558

[39] Ahearn JM Fearon DT. Structure and function of the complement receptors, CR1 (CD35) and CR2 (CD21). Adv Immunol (1989) 46:183–219. doi: 10.1016/S0065-2776(08)60654-9 2551147

[40] Peerschke EI Reid KB Ghebrehiwet B. Platelet activation by C1q results in the induction of alpha IIb/beta 3 integrins (GPIIb-IIIa) and the expression of P-selectin and procoagulant activity. J Exp Med (1993) 178(2):579–87. doi: 10.1084/jem.178.2.579 PMC2191135 7688027

[41] Krarup A Wallis R Presanis JS Gál P Sim RB. Simultaneous activation of complement and coagulation by MBL-associated serine protease 2. PloS One (2007) 2(7):e623–3. doi: 10.1371/journal.pone.0000623 PMC1910608 17637839

[42] Dalakas MC Alexopoulos H Spaeth PJ. Complement in neurological disorders and emerging complement-targeted therapeutics. Nat Rev Neurol (2020) 16(11):601–17. doi: 10.1038/s41582-020-0400-0 PMC7528717 33005040

[43] Roth A Barcellini W D’Sa S Miyakawa Y Broome CM Michel M. Complement C1s inhibition with sutimlimab results in durable response in cold agglutinin disease: CARDINAL study 1-year interim follow-up results. Haematologica (2022) 107(7):1698–702. doi: 10.3324/haematol.2021.279812 PMC9244812 35172561

[44] Neth O Jack DL Dodds AW Holzel H Klein NJ Turner MW. Mannose-binding lectin binds to a range of clinically relevant microorganisms and promotes complement deposition. Infect Immun (2000) 68(2):688–93. doi: 10.1128/IAI.68.2.688-693.2000 PMC97193 10639434

[45] Merle NS Church SE Fremeaux-Bacchi V Roumenina LT. Complement system part I - molecular mechanisms of activation and regulation. Front Immunol (2015) 6(JUN):1–30. doi: 10.3389/fimmu.2015.00262 26082779 PMC4451739

[46] Endo Y Nakazawa N Iwaki D Takahashi M Matsushita M Fujita T. Interactions of ficolin and mannose-binding lectin with fibrinogen/fibrin augment the lectin complement pathway. J Innate Immun (2009) 2(1):33–42. doi: 10.1159/000227805 20375621

[47] Jenny L Dobó J Gál P Schroeder V. MASP-1 induced clotting - the first model of prothrombin activation by MASP-1. PloS One (2015) 10(12):1–13. doi: 10.1371/journal.pone.0144633 PMC4672900 26645987

[48] Krarup A Gulla KC Gál P Hajela K Sim RB. The action of MBL-associated serine protease 1 (MASP1) on factor XIII and fibrinogen. Biochim Biophys Acta (2008) 1784(9):1294–300. doi: 10.1016/j.bbapap.2008.03.020 18456010

[49] Hess K Ajjan R Phoenix F Dobó J Gál P Schroeder V. Effects of MASP-1 of the complement system on activation of coagulation factors and plasma clot formation. PloS One (2012) 7(4):e35690. doi: 10.1371/journal.pone.0035690 22536427 PMC3335018

[50] Takahashi K Chang W-C Takahashi M Pavlov V Ishida Y La Bonte L. Mannose-binding lectin and its associated proteases (MASPs) mediate coagulation and its deficiency is a risk factor in developing complications from infection, including disseminated intravascular coagulation. Immunobiology (2011) 216(1–2):96–102. doi: 10.1016/j.imbio.2010.02.005 20399528 PMC2912947

[51] Pavlov VI Tan YS McClure EE La Bonte LR Zou C Gorsuch WB. Human mannose-binding lectin inhibitor prevents myocardial injury and arterial thrombogenesis in a novel animal model. Am J Pathol (2015) 185(2):347–55. doi: 10.1016/j.ajpath.2014.10.015 PMC4305178 25482922

[52] Hillmen P Muus P Dührsen U Risitano AM Schubert J Luzzatto L. Effect of the complement inhibitor eculizumab on thromboembolism in patients with paroxysmal nocturnal hemoglobinuria. Blood (2007) 110(12):4123–8. doi: 10.1182/blood-2007-06-095646 17702897

[53] Ueda Y Mohammed I Song D Gullipalli D Zhou L Sato S. Murine systemic thrombophilia and hemolytic uremic syndrome from a factor H point mutation. Blood (2017) 129(9):1184–96. doi: 10.1182/blood-2016-07-728253 PMC5374733 28057640

[54] Pryzdial ELG Leatherdale A Conway EM. Coagulation and complement: Key innate defense participants in a seamless web. Front Immunol (2022) 13:918775. doi: 10.3389/fimmu.2022.918775 36016942 PMC9398469

[55] Feng S Liang X Cruz MA Vu H Zhou Z Pemmaraju N. The interaction between factor H and Von Willebrand factor. PloS One (2013) 8(8):e73715. doi: 10.1371/journal.pone.0073715 23991205 PMC3753316

[56] Fung M Loubser PG Ündar A Mueller M Sun C Sun WN. Inhibition of complement, neutrophil, and platelet activation by an anti-factor D monoclonal antibody in simulated cardiopulmonary bypass circuits. J Thorac Cardiovasc Surg (2001) 122(1):113–22. doi: 10.1067/mtc.2001.114777 11436043

[57] Yaspan BL Williams DF Holz FG Regillo CD Li Z Dressen A. Targeting factor D of the alternative complement pathway reduces geographic atrophy progression secondary to age-related macular degeneration. Sci Transl Med (2017) 9(395):eaaf1443. doi: 10.1126/scitranslmed.aaf1443 28637922

[58] Aiello S Gastoldi S Galbusera M Ruggenenti P Portalupi V Rota S. C5a and C5aR1 are key drivers of microvascular platelet aggregation in clinical entities spanning from aHUS to COVID-19. Blood Adv (2022) 6(3):866–81. doi: 10.1182/bloodadvances.2021005246 PMC8945302 34852172

[59] Shivshankar P Li Y-D Mueller-Ortiz SL Wetsel RA. In response to complement anaphylatoxin peptides C3a and C5a, human vascular endothelial cells migrate and mediate the activation of B-cells and polarization of T-cells. FASEB J . (2020) 34(6):7540–60. doi: 10.1096/fj.201902397R PMC11905332 32301538

[60] Polley MJ Nachman RL. Human platelet activation by C3a and C3a des-arg. J Exp Med (1983) 158(2):603–15. doi: 10.1084/jem.158.2.603 PMC2187348 6604123

[61] Landsem A Emblem Å Lau C Christiansen D Gerogianni A Karlsen BO. Complement C3b contributes to Escherichia coli-induced platelet aggregation in human whole blood. Front Immunol (2022) 13:1020712. doi: 10.3389/fimmu.2022.1020712 36591264 PMC9797026

[62] MannesMPechtlVHafnerSDoplerAErikssonOManivelVA. Complement & platelets: Prothrombotic cell activation requires membrane attack complex induced release of danger signals. Blood Adv (2023). doi: 10.1182/bloodadvances.2023010817 37428869

[63] Gushiken FC Han H Li J Rumbaut RE Afshar-Kharghan V. Abnormal platelet function in C3-deficient mice. J Thromb Haemost. (2009) 7(5):865–70. doi: 10.1111/j.1538-7836.2009.03334.x PMC2867673 19291167

[64] Kim DD Song W-C. Membrane complement regulatory proteins. Clin Immunol (2006) 118(2–3):127–36. doi: 10.1016/j.clim.2005.10.014 16338172

[65] Liszewski MK Farries TC Lublin DM Rooney IA Atkinson JP. Control of the complement system. Adv Immunol (1996) 61:201–83. doi: 10.1016/S0065-2776(08)60868-8 8834497

